# A Bibliometric Analysis of Research on Temporomandibular Joint Disc Displacement from 1992 to 2022

**DOI:** 10.3390/healthcare11142108

**Published:** 2023-07-24

**Authors:** Qiuhao Wang, Jin Jia, Changhan Zhou, Wang Ye, Ruiye Bi

**Affiliations:** State Key Laboratory of Oral Diseases, National Clinical Research Center for Oral Diseases, West China Hospital of Stomatology, Sichuan University, Chengdu 610041, China

**Keywords:** temporomandibular joint disc displacement, orthognathic surgery, temporomandibular joint, bibliometric analysis, hotspots

## Abstract

The temporomandibular joint (TMJ) disc displacement is the most common temporomandibular disorders (TMD) condition. It causes clicking, pain, limited mandibular movements, and even masticatory difficulties in many people. The aim of this study is showcasing hotspots and frontiers in the field and providing a reference for the future research by a bibliometric analysis. Studies published from 1992 to 2022 were retrieved from Web of Science Core Collection on 23 April 2023. A total of 1882 studies (1739 articles and 143 reviews) were included in the bibliometric analysis. From 1992 to 2022, the annual number of publications and citations greatly increased. The United States of America (USA) contributed the most publications about TMJ disc displacement. Shanghai Jiao Tong University was the most productive institution; meanwhile, Yang, C. from this institution was the most prolific author. The University of Washington was the most influential institution, and Brooks, S. was the most influential author. Diagnostic criteria and management of TMJ disc displacement, as well as TMJ disc displacement-associated conditions, might be a hotspot for current global research. We provided an objective and valuable reference for future research on TMJ disc displacement.

## 1. Introduction

The temporomandibular joint (TMJ) is the sole mobile joint located in the maxillofacial region, necessary for functional activities including chewing, swallowing, and speaking [1]. The TMJ is mainly composed of the articular condyle, articular disc, articular fossa, fibrous capsule, and synovial membrane [2]. Temporomandibular disorders (TMD) are a group of musculoskeletal disorders that affect the TMJ and associated structures, encompassing masticatory muscle disorders, TMJ structure abnormalities, inflammatory diseases, and osteoarthropathy [3]. TMJ disc displacement is one of the most common TMD conditions, which mainly results from orthopedic instability in the joint [4]. Additionally, trauma, excessive movements of the jaw, and ill-suited condyle morphology can also contribute towards TMJ disc displacement [5]. The incidence of TMJ disc displacement in TMD patients ranges from 38% to 73%, while the general population incidence ranges between 18% and 35% [6]. Some studies have shown that TMJ disc displacement is more likely to occur in adolescents, which has a significant relationship with sexual maturation [7]. Compared with posterior, medial, or lateral displacement, anterior disc displacement is more commonly encountered clinically [8]. Magnetic resonance imaging (MRI) is currently the most accurate and essential diagnostic modality for determining the position of the articular disc [9]. TMJ disc displacement can be classified as disc displacement with reduction (DDwR) or disc displacement without reduction (DDwoR) [10]. In DDwR, which holds the higher frequency, there exists an abnormal disc–condyle relationship [11]. The displaced disc returns to the normal position on mouth opening, thereby producing a reciprocal click [12]. In DDwoR, the disc remains displaced in relation to the condyle, whether the mouth is open or closed [13]. This typically results in pain, joint sound, limited movement, and even condylar degeneration [14]. Currently, the management of TMJ disc displacement seeks to alleviate pain during function and to attain a standard range of mandibular movements [15]. Conservative measures include medication, physical therapy, splint therapy, etc., while surgical options comprise arthrocentesis, arthroscopy, and open joint surgeries, among others [16].

With the advancement of medical research and technology, patients suffering from TMJ disc displacement can now receive effective treatment [17]. Although research on this condition has been relatively developed, there still exists some unclear aspects, such as the correlation between osteoarthrosis and TMJ disc displacement [18]. Due to the long research interval, vast amount of data, varying quality of scientific publications, and unnecessary redundancy, researchers are compelled to dedicate considerable time in sifting through and identifying relevant literature in related fields [19], which may impede the progress of TMJ disc displacement research. Accordingly, an analysis and summary of knowledge structure, research centers, and frontiers could assist researchers [20]. Despite bibliometric studies having been conducted on TMD [21], there is a dearth of bibliometric analysis regarding TMJ disc displacement.

Bibliometrics is a method for assessing and monitoring the progress of specific disciplines via statistical analysis of published data [22]. Bibliometric analysis can be used to determine the outputs and citations of authors, institutions, and countries and the keyword frequency of research hotspots and frontiers in particular fields [23]. It plays a pivotal role in assisting relevant individuals in categorizing research trajectories, unearthing disciplinary frontiers, and identifying research hotspots [24]. This study aims to conduct an extensive bibliometric analysis of publications concerning TMJ disc displacement, with the goal of identifying research hotspots and potential trends and subsequently providing valuable reference guidelines for future studies.

## 2. Methods and Materials

### 2.1. Source of Literature and Search Strategies

The literature data of this study were retrieved from Web of Science Core Collection (WoSCC) on 23 April 2023. The following keywords were used for all relevant studies: TS = (“temporomandibular joint *” OR “temporomandibular articular *” OR TMJ) AND TS = ( disc OR discs OR disk OR disks) AND TS = (displacement OR dislocation). CiteSpace (version 6.2.R2 [advanced]) was applied to retain articles and reviews published from 1 January 1992 to 31 December 2022, while corrections, editorial materials, letters, meeting abstracts and proceeding papers were removed.

### 2.2. Data Analysis

VOSviewer (version 1.6.19) was a software tool for constructing and visualizing bibliometric networks [25]. In the visual maps, the nodes represent authors, institutions, countries, journals, papers, and keywords; the lines represent co-occurrence or co-citation; and the sizes of the nodes were determined by the frequency of occurrence while the thickness of the lines was determined by the strength of co-occurrence relationship. In the clustering maps, having a common theme was given the same color. In the other maps, elements were given different colors based on the average year of the occurrence, with blue and green elements appearing earlier and yellow and red elements appearing later.

CiteSpace was an Java application for data analysis and visualization and identifying the burst keywords and the burst references [26]. A burst represents a great increase in the interest of a researcher which could predict the academic trends and hotspots in TMJ disc displacement. All software was running on Windows 11.

## 3. Results

### 3.1. Analysis of General Research Trends

**Figure 1:**
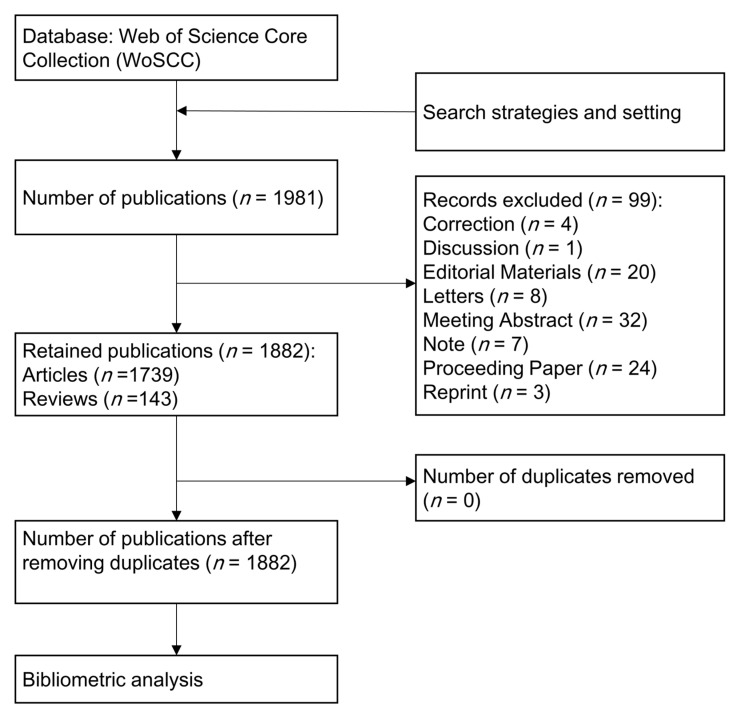
Flowchart for the search and selection process of this a bibliometric analysis.

**Figure 2:**
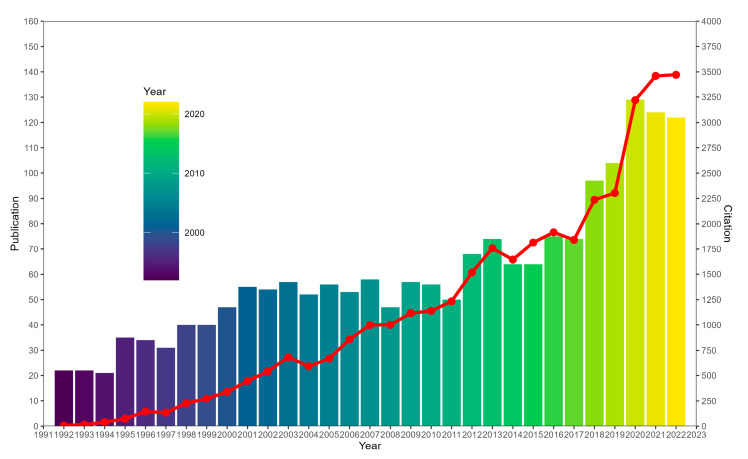
Analysis of global research trend of TMJ disc displacement from 1992 to 2022.

After removing duplicates and publications that did not meet the type requirements, a total of 1882 publications (1739 articles and 143 reviews) were finally included in bibliometric analysis (Figure 1). R package “ggplot2” was used to analyze the general research trends (such as number of publications per year and number of citations per year [27]. As shown in Figure 2, over the past three decades, there was an overall upward trend in the number of publications and citations regarding TMJ disc displacement although there were small fluctuations. It could be specifically divided into three stages: in 1992–2001, the average number of publications per year was 34.7 while the average number of citations per year was 169.9; in 2002–2011, the number of publications and citations per year were 54 and 882.9; in the period 2012–2022, these two numbers have spiked to 90.5 and 2289.3. In each of the three time periods, the years with the highest number of publications were 2001 (with 55 publications), 2007 (with 58 publications), and 2020 (with 129 publications). And the annual number of publications and citations has grown from 22 and 7 in 1992 to 122 and 3471 in 2022 (Table S1).

Notably, the annual number of publications exceeded 100 from 2019 to 2022, with an average of 119.75, and the number of publications peaked in 2020 (*n* = 129). The annual number of publications and citations showed surges in 2019–2020, which indicated that TMJ disc displacement-related research was attracting the attention of researchers worldwide in recent years.

### 3.2. Analysis of the Cooperative Relationship

#### 3.2.1. Countries

**Figure 3:**
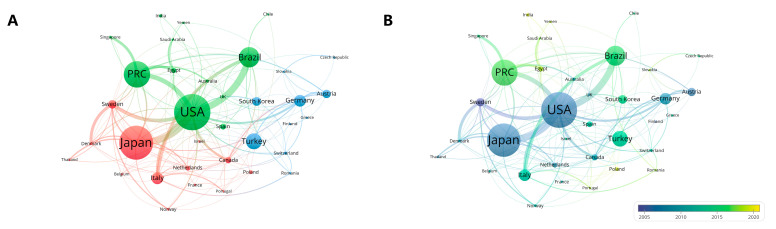
(**A**)Visualization graphs of clustering analysis of countries and (**B**) visualization graph of countries with timeline view.

In total, 71 countries had published studies on TMJ disc displacement, with 38 countries publishing more than five studies (Table S2). The evolution of the volume of articles published in these 38 countries was shown in Figure S1. The top ten countries in terms of number of publications were the main sources of TMJ disc displacement-related publications, accounting for 1572 (83.53% of total publications) (Table 1). Overall, the countries with most publication was the United States of America (USA) (*n* = 314), indicating the USA was the main research forces in the field of TMJ disc displacement research. Moreover, from 1992 to 1996, the USA contributed the most publications; from 1997 to 2011, Japan contributed the most relevant studies; from 2012 to 2022, the PRC had a dramatic increase in the volume of published literature, turning it to the leading source of research in the field (Figure S1). And the country with the strongest impact studies was Sweden, with an average of 35.33 citations per study. Through cluster analysis and visualization by VOSviewer (Figure 3A), it can be divided into three clusters, one of which was centered in the USA and one was centered in Japan. The researchers of the USA had a great deal of cooperation with other countries, such as Japan, the People′s Republic of China (PRC), and Brazil. And we found that Sweden, Finland, and the USA started earlier in this research field, while Yemen, Poland, and India started later (Figure 3B).

#### 3.2.2. Institutions

**Figure 4:**
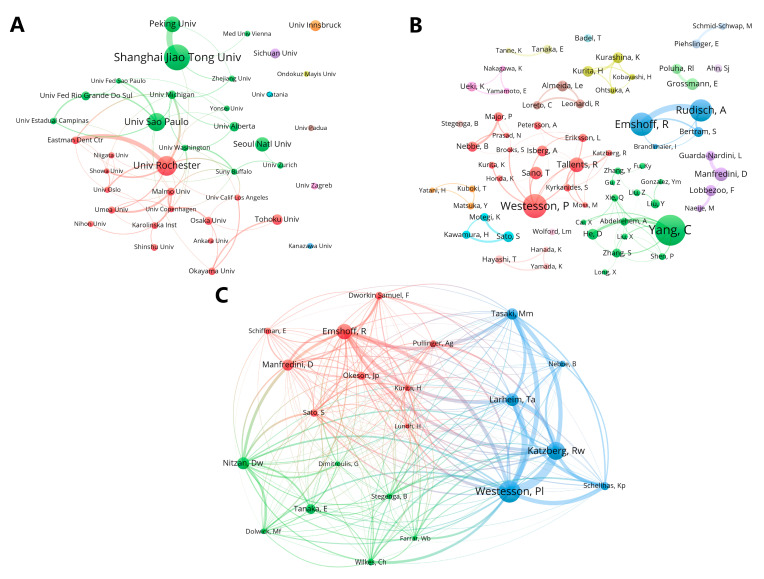
Clustering analysis of (**A**) institutions and (**B**) authors and (**C**) visualization graph of co-citation analysis of authors.

A total of 1356 institutions had published relevant literature, with 38 of them publishing more than 15 publications. The studies of the University of Washington were the most influential, reaching an average of 86.61 citations per article, although it was not one of the top 10 institutions with the most publications. Shanghai Jiao Tong University, the University of Rochester, and the University of São Paulo contributed the most publications on TMJ disc displacement (Table 2), showing that these institutions had a strong research capacity in this field. As shown in Figure 4A, Shanghai Jiao Tong University and Peking University had close collaboration.

#### 3.2.3. Authors

For the authors, 68 authors published more than 10 studies on TMJ disc displacement, and a total of 5192 authors had conducted research on this topic. The top 10 most productive authors were listed in Table 3, and Yang, C. from Shanghai Jiao Tong University contributed the most publications (*n* = 54). Figure 4B indicates that Yang, C. (green), Westesson, P. (red), Emshoff, R., and Rudisch, A. (blue) were the centers of their respective clusters. According to citations per study, Brooks, S. was the most influential researcher in this field. Based on a co-citation map (Figure 4C), it could be divided into three clusters, and we found that Westesson, P. and Katzberg, R. might have similar research themes. We also noted that the average publication time of Westesson, P., Tallents, R., and Isberg, A. was from 1992 to 2001, indicating their early start in this field; the average publication year of Yang, C., Manfredini, D., and Grossmann, E. was from 2012 to 2022, showing that they have been highly active in this field in recent years; and the rest of the top 10 authors (Emshoff, R., Rudisch, A., Sano, T., and Lobbezoo, F.) were active in 2002–2011 based on the average year of their publications.

### 3.3. Analysis of Journals and Highly Cited Papers

#### 3.3.1. Journals

**Figure 5:**
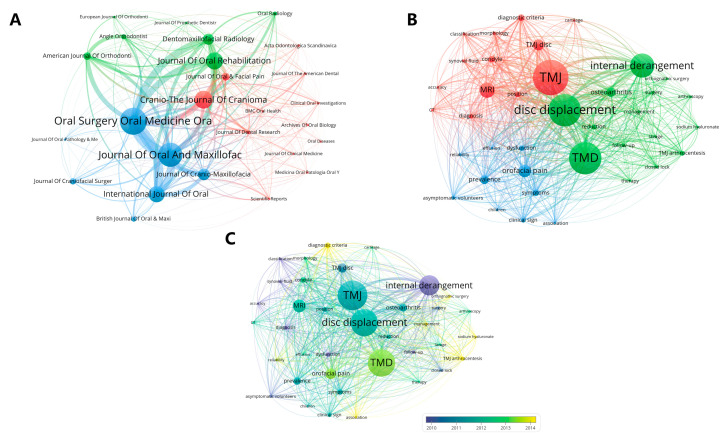
Clustering analysis of (**A**) journals and (**B**) keywords and (**C**) visualization graph of keywords with timeline view.

A total of 322 journals have published research on TMJ disc displacement, with 27 journals publishing more than 10 publications. These 10 journals published 51.7% of the publications (*n* = 973) in the field of TMJ disc displacement research. The most active source journals by the count of documents were *Oral Surgery Oral Medicine Oral Pathology, and Oral Radiology (n = 181)*, *Journal of Oral and Maxillofacial Surgery (n = 162)*, and *Cranio-the Journal of Craniomandibular & Sleep Practice (n = 120)* (Table 4). The *Journal of Dental Research* was the most influential journal in TMJ disc displacement, with an average 23.58 citations per paper. The evolution of the volume of articles published in these top 10 journals is shown in Figure S2. From 1992 to 2011, *Oral Surgery Oral Medicine Oral Pathology, and Oral Radiology* published the most studies in this field, while the *Journal of Cranio-Maxillofacial Surgery* published the most from 2012 to 2016, and *Cranio-The Journal of Craniomandibular & Sleep Practice* published the most from 2017 to 2022. Also, we found that the most co-cited journals were *Oral Surgery Oral Medicine Oral Pathology*, *and Oral Radiology* and the *Journal of Oral and Maxillofacial Surgery*, indicating some similarity between the studies on TMJ disc displacement published in these two journals (Figure 5A).

#### 3.3.2. Highly Cited Papers

The three most cited papers were “Degenerative disorders of the temporomandibular joint: etiology, diagnosis, and treatment” by Tanaka E. et al., published in the *Journal of Dental Research* in 2008 [28], “Research diagnostic criteria for temporomandibular disorders (RDC/TMD): development of image analysis criteria and examiner reliability for image analysis” by Ahmad M. et al., published in *Oral Surgery Oral Medicine Oral Pathology*, *and Oral Radiology* in 2009 [29], and “Classification and prevalence of temporomandibular joint disc displacement in patients and symptom-free volunteers” by Tasaki M.M. et al., published in the *American Journal of Orthodontics and Dentofacial Orthopedics* in 1996 (Table 5) [30]. Tanaka E et al.′s study provided a detailed review of the etiology of TMJ-osteoarthrosis, the importance in the diagnosis, and the non-invasive and invasive modalities utilized in TMJ-osteoarthrosis management [28]. Ahmad M et al.′s study is a part of the Multisite Research Diagnostic Criteria for Temporomandibular Disorders (RDC/TMD) Validation Project, and this study developed comprehensive image analysis criteria for the RDC/TMD Validation Project, which could be applied for assessing osteoarthritis using CT and for TMJ disc position and effusion using MRI [29], and the study of Tasaki MM et al. developed a classification system for TMJ disc displacement and evaluated the prevalence of the various types of TMJ disc displacement in patients and symptom-free volunteers [30].

### 3.4. Analysis of Keywords

We identified a total of 3703 keywords, of which those appearing more than 50 times were plotted in the clustering map (Figure 5B). These keywords were classified into three clusters. The cluster in red is mainly related to the diagnosis classification of TMJ disc displacement, including the keywords such as MRI, CT, classification, diagnosis criteria, accuracy, etc. The cluster in green mainly represents the treatments for TMJ disc displacement and prognosis after therapy, including keywords such as management, orthognathic surgery, lavage, TMJ arthrocentesis, follow-up, etc. And the blue cluster is mainly related to clinic epidemiology of TMJ disc displacement, such as prevalence, children, asymptomatic volunteers, etc. Meanwhile, based on the occurrence time of keywords (Figure 5C), we found that the classification and dysfunction of TMJ disc displacement began to be studied earlier while the diagnostic criteria and management of TMJ disc displacement were being intensively studied in recent years.

**Figure 6:**
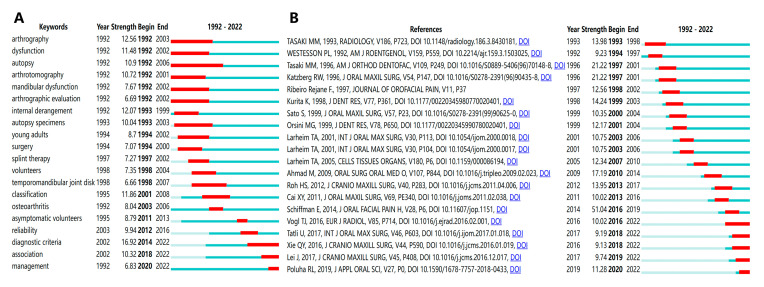
(**A**) The top 20 keywords with the strongest burst. (**B**) The top 20 references with the strongest burst.

The top 20 strongest burst keywords are shown in Figure 6A. From 1992 to 2001, the strongest burst keywords were “arthrography”, “internal derangement”, and “classification”. From 2002 to 2011, the burst keywords were “osteoarthritis” and “asymptomatic volunteers”. From 2012 to 2022, the newest three burst keywords are “diagnostic criteria”, “association”, and “management”. It indicates that research themes are beginning to shift toward diagnostic criteria and management of TMJ disc displacement, and these might be the hotspots for current global research.

### 3.5. Analysis of References

The top 20 strongest burst references are shown in Figure 6B. The three strongest burst references are the following: “Diagnostic Criteria for Temporomandibular Disorders (DC/TMD) for Clinical and Research Applications: recommendations of the International RDC/TMD Consortium Network and Orofacial Pain Special Interest Group” by Schiffman E. et al. (2014), which recommended a new evidence-based DC/TMD protocol for use in both clinical and research settings [31]; “Classification and prevalence of temporomandibular joint disk displacement in patients and symptom-free volunteers” by Tasaki M.M. et al. (1996), which was also one of the most cited studies in this research field [30]; and “Anatomic disorders of the temporomandibular joint disc in asymptomatic subjects” by Katzberg R.W. et al. (1996), which found that the incidence of TMJ disc displacement was significantly higher in symptomatic subjects than in asymptomatic volunteers and bruxing was statistically linked to TMJ disc displacement [32].

The following recent publications were identified as references with burst, showing they were generating widespread interest. They were “Temporomandibular joint disc displacement with reduction: a review of mechanisms and clinical presentation” by Poluha R.L. et al. (2019) [33], “Degenerative temporomandibular joint changes associated with recent-onset disc displacement without reduction in adolescents and young adults” by Lei J. et al. (2017) [34], and “Will unilateral temporomandibular joint anterior disc displacement in teenagers lead to asymmetry of condyle and mandible? A longitudinal study” by Xie Q. et al. (2016) [35].

## 4. Discussion

In the present study, bibliometric analysis was carried out on publications related to TMJ disc displacement between 1992 and 2022. The annual count of publications and citations in 2022 witnessed a remarkable surge compared to those in 1992. The USA, Japan, PRC, Brazil, and Turkey held a leading position in this research area, though international cooperation still requires greater strengthening. Among the top 10 productive institutions, three are located in the PRC; two are located in Brazil; and one each are located in the USA, South Korea, Japan, Canada, and Austria. Notably, the University of Rochester from the USA emerged as the most influential of the top 10 institutions, with an average citation rate of 53.7 per study. Yang, C. from Shanghai Jiao Tong University was identified as the world′s most prolific author, with research interests spanning TMJ surgery, minimally invasive surgery, integrated alveolar surgery, and odontogenic stem cells. *Oral Surgery Oral Medicine Oral Pathology*, *and Oral Radiology* published the most studies on TMJ disc displacement, and this journal covered topics including current issues such as dental implants, treatment of HIV-infected patients, and the evaluation and treatment of TMJ disorders. Among the 10 most active source journals, the *Journal of Oral & Facial Pain and Headache*, the *American Journal of Orthodontics and Dentofacial Orthopedics*, and *Oral Surgery Oral Medicine Oral Pathology, and Oral Radiology* had a relatively high average citation rate. Thus, researchers interested in publishing TMJ disc displacement studies could give priority to these three journals.

### 4.1. Burst References

The top three most recent references with burst were all related to the recommendations for the management of TMJ disc displacement, specifically Poluha R.L. et al. [33] publishing “Temporomandibular joint disc displacement with reduction: a review of mechanisms and clinical presentation” in 2019, which suggested that management should be conducted when DDwR is the patient’s main complaint and when the noise motivated the patient to seek management and/or the click is accompanied by pain. Although DDwR is a highly prevalent clinical condition, it still raised many issues, such as the risk of progression and how to manage it effectively, which requires to further investigation. Lei J. et al. [34] published “Degenerative temporomandibular joint changes associated with recent-onset disc displacement without reduction in adolescents and young adults” in 2017 and found an association between the onset of TMJ disc displacement and degenerative TMJ changes. The risk of developing early-stage osteoarthritis (OA) changes increased appreciably 1 month after the onset of TMJ disc displacement. Early diagnosis and intervention of TMJ disc displacement was therefore recommended, especially in children and adolescents [36]. “Will unilateral temporomandibular joint anterior disc displacement in teenagers lead to asymmetry of condyle and mandible? A longitudinal study” by Xie Q. et al. [35] in 2016 observed the progress of unilateral juvenile anterior disc displacement (UJADD) during natural course and analyzed its effect over symmetry of the mandible. The results suggested that UJADD results in asymmetric growth bilateral TMJs, especially condylar height, which was much shorter on the ipsilateral side. At the same time, mandibular asymmetry became worse during the natural course of UJADD.

### 4.2. Burst Keywords

In recent years, three keywords with burst related to TMJ disc displacement have emerged: diagnostic criteria, association, and management.

#### 4.2.1. Diagnostic Criteria

Some studies related to diagnostic criteria presented the evidence-based new Axis I and Axis II Diagnostic Criteria for TMD (DC/TMD) to be used in both clinical and research settings and presented the processes related to their development [37]. Dworkin and LeResche developed the Research Diagnostic Criteria for TMD (RDC/TMD) criteria for the standardization of the diagnostic methodology for most common TMD in 1992 [29]. It was an important advancement in providing a diagnostic system to guide research into TMD. In 2014, the criteria were revised to become the DC/TMD with specific diagnostic TMD subsets [38]. For the diagnosis of the TMJ disc displacement, DC/TMD had advocated history, clinical examination, and imaging as the standard approach for the diagnosis of the TMJ disc displacement [39]. MRI was considered the most effective imaging tool (“gold standard”) for diagnosis of the TMJ disc displacement [40]. However, MRI could only determine if the disc was displaced partially or completely with mouth closed and if the disc partially or completely reduced on opening, not whether this causes pain or limited opening, which might affect the validation of a clinical diagnosis [38]. Some researchers suggested that DC/TMD could be identified as simple reliable economical tool for the diagnosis of TMJ disc displacement [38]. Moreover, we still have the need for an improved technique by which to confirm TMJ disc displacement.

#### 4.2.2. Association

TMJ disc displacement was associated with numerous signs and symptoms [41]. It had been observed that TMJ disc displacement was able to produce pain, dysfunction, and bone changes in the joint surfaces [42]. Numerous studies demonstrated a high prevalence of disc displacement in patients with TMJ pain and dysfunction, but some studies had also shown that TMJ disc displacement may present without any pain [43]. What is more, the researchers confirmed the positive correlation between some specific genes and a higher risk of articular disc displacement, such as COL5A1 rs12722 [44]. TMJ disc displacement was frequently accompanied by OA, and OA significantly altered the bone quality of the condyle [45], which might be relevant to MMP overexpression [46]. In clinical practice, some research showed that joint effusions were frequently present in painful joints with TMJ disc displacement but rarely in normal joints [47]. Joint effusions were commonly attributed to OA and can often be considered an indication for TMJ surgery [40]. However, research on this field remains unclear and controversial [48], warranting further investigation to determine the association between TMJ disc displacement and other diseases.

#### 4.2.3. Management

A lot of research focused on the management of DDwoR [49]. Conservative treatments, such as splint therapy, represented the primary management for DDwoR [50]. If there was no improvement with conservative methods, arthrocentesis was generally considered as a second-step therapy [51]. Although splint therapy was widely used and appears to be effective, the precise mechanisms of action remained controversial [41]. Some researchers have raised the possibility of surgical methods as the initial management [52]. Additionally, patients diagnosed with severe TMJ disc displacement might require an interdisciplinary approach to the treatment and should be referred to maxillofacial surgeons and even rheumatologists [53]. However, there is currently no consensus on the optimal therapy for TMJ disc displacement, which is still a challenge for surgeons who treat various TMJ disorders [54].

## 5. Limitations

This study has several limitations. First, since all the literature was collected solely from WOSCC, there is a possible risk of missing studies included in other databases. Second, due to software limitations of CiteSpace and VOSviewer, self-citations could not be excluded, potentially leading to bias. Lastly, since some excellent newly published articles may have been omitted due to lag, there is a possibility that the study′s findings are not up to date.

## 6. Conclusions

This is the first bibliometric analysis of TMJ disc displacement. Over the past three decades, there has been a growing amount of research on TMJ disc displacement. The USA, Japan, and PRC are the core contributor in this field. Research in recent years has focused on diagnostic criteria and management of TMJ disc displacement, and researchers should be concerned about TMJ disc displacement-associated conditions such as OA and joint effusions. In the future, investigations should focus on the refinement of diagnostic criteria and treatment modalities concerning TMJ disc displacement, with the ultimate goal of achieving transformative advancements.

## Figures and Tables

**Table 1:** Analysis of top 10 countries with most relevant publication in TMJ disc displacement.

Rank	Country	Documents	Citations	Average Citations	Leading Contributors (Number of Publications)
1	USA	314	9853	31.38	Per-Lennart Westesson (42); Ross H Tallents (26)
2	Japan	293	5983	20.42	Tsukasa Sano (22); Kenji Kurashina (19)
3	PRC	227	2520	11.1	Chi Yang (54); Danqing He (19)
4	Brazil	175	2617	14.95	Eduardo Grossmann (21); Rodrigo Lorenzi Poluha (18)
5	Turkey	137	1727	12.61	Burcu Bas (9); Kaan Orhan (7)
6	Italy	103	1965	19.08	Daniele Manfredini (24); Luca Guarda-Nardini (17)
7	Germany	102	1917	18.79	Peter Rammelsberg (12); Mike T John (12)
8	South Korea	77	1074	13.95	Sug-Joon Ahn (15); Tae-Woo Kim (9)
9	Sweden	72	2544	35.33	Annika Isberg (19); Lars Eriksson (15)
10	Austria	72	1866	25.92	Ruediger Emshoff (41); Ansgar Rudisch (39)

**Table 2:** Analysis of top 10 productive institutions in TMJ disc displacement.

Rank	Institution	Documents	Citations	Average Citations	Countries
1	Shanghai Jiao Tong University	71	784	11.04	PRC
2	University of Rochester	54	2900	53.7	USA
3	University of São Paulo	50	715	14.3	Brazil
4	Peking University	43	426	9.91	PRC
5	Seoul National University	39	674	17.28	South Korea
6	University of Innsbruck	30	1180	39.33	Austria
7	Tohoku University	29	541	18.66	Japan
8	Sichuan University	27	244	9.04	PRC
9	University of Alberta	26	876	33.69	Canada
10	Universidade Federal do Rio Grande do Sul	25	223	8.92	Brazil

**Table 3:** Analysis of top 10 prolific authors in TMJ disc displacement.

Rank	Author	Documents	Citations	Average Publication Year	Average Citations	Affiliated Institution
1	Chi Yang	54	707	2016	13.09	Shanghai Jiao Tong University
2	Per-Lennart Westesson	42	2821	1996	67.17	University of Rochester
3	R. Diger Emshoff	41	1365	2005	33.29	Medical University of Innsbruck
4	Ansgar Rudisch	39	1304	2005	33.44	Medical University of Innsbruck
5	Ross H. Tallents	26	1511	1999	58.12	University of Rochester
6	Daniele Manfredini	24	713	2013	29.71	University of Siena
7	Tsukasa Sano	22	614	2005	27.91	The Scripps Research Institute
8	Eduardo Grossmann	21	217	2019	10.33	Universidade Federal do Rio Grande do Sul
9	Frank Lobbezoo	20	531	2011	26.55	University of Amsterdam and Vrije Universiteit Amsterdam
10	Annika Isberg	19	829	2001	43.63	Karolinska Institutet

**Table 4:** Analysis of top 10 journals published most relevant publication on TMJ disc displacement.

Rank	Journal	Documents	Citations	Average Publication Year	Average Citations	IF (2022)
1	*Oral Surgery Oral Medicine Oral Pathology*, *and Oral Radiology*	181	5436	2005	30.03	2.9
2	*Journal of Oral and Maxillofacial Surgery*	162	4324	2008	26.69	1.9
3	*Cranio-The Journal of Craniomandibular & Sleep Practice*	120	1331	2010	11.09	1.6
4	*Journal of Oral Rehabilitation*	115	2216	2010	19.27	2.9
5	*International Journal of Oral and Maxillofacial Surgery*	108	2585	2010	23.94	2.4
6	*Journal of Cranio-Maxillofacial Surgery*	78	1216	2015	15.59	3.1
7	*Dentomaxillofacial Radiology*	75	1509	2009	20.12	3.3
8	*Journal of Oral & Facial Pain and Headache*	51	1765	2008	34.61	2.5
9	*American Journal of Orthodontics and Dentofacial Orthopedics*	50	1803	2004	36.06	3.0
10	*Journal of Craniofacial Surgery*	43	331	2015	7.7	0.9

**Table 5:** Analysis of top 10 papers with most citations in TMJ disc displacement.

Rank	Title	Source Journals	Citations	Publication Year
1	Degenerative Disorders of the Temporomandibular Joint: Etiology, Diagnosis, and Treatment	*Journal of Dental Research*	459	2008
2	Research diagnostic criteria for temporomandibular disorders (RDC/TMD): development of image analysis criteria and examiner reliability for image analysis	*Oral Surgery Oral Medicine Oral Pathology and*, *Oral Radiology*	369	2009
3	Classification and prevalence of temporomandibular joint disk displacement in patients and symptom-free volunteers	*American Journal of Orthodontics and Dentofacial Orthopedics*	257	1996
4	Anatomic disorders of the temporomandibular joint disc in asymptomatic subject	*Journal of Oral and Maxillofacial Surgery*	229	1996
5	Prevalence of temporomandibular disorder subtypes, psychologic distress, and psychosocial dysfunction in Asian patients	*Journal of Oral & Facial Pain and Headache*	226	2003
6	Temporomandibular joint: relationship between MR evidence of effusion and the presence of pain and disk displacement.	*American Journal of Roentgenology*	209	1992
7	Imaging of the temporomandibular joint: A position paper of the American Academy of Oral and Maxillofacial Radiology	*Oral Surgery Oral Medicine Oral Pathology, and Oral Radiology*	185	1997
8	Temporomandibular disorders: Old ideas and new concepts	*Cephalalgia*	166	2017
9	A Systematic Review of the Effectiveness of Exercise, Manual Therapy, Electrotherapy, Relaxation Training, and Biofeedback in the Management of Temporomandibular Disorder	*Physical Therapy & Rehabilitation Journal*	164	2006
10	Changes in temporomandibular joint dysfunction after orthognathic surgery	*Journal of Oral and Maxillofacial Surgery*	154	2003

## Data Availability

The original data presented in the study are included in the article and Supplementary Materials, and further inquiries can be directed to the corresponding author.

## Supplementary Materials

The following supporting information can be downloaded at: https://www.mdpi.com/article/10.3390/healthcare11142108/s1, Figure S1: Visualization graph of the evolution in (**A**) rank 1–10 countries, (**B**) rank 11–20 countries, (**C**) rank 21–30 countries, and (**D**) rank 31–38 countries based on the number of publications; Figure S2: Visualization graph of the evolution of the volume of publications in top 10 journals; Figure S3: Visualization graph of institutions with timeline view; Figure S4: Visualization graph of authors with timeline view; Figure S5: Visualization graph of journals with timeline view; Table S1: Number of publications and citations in TMJ disc displacement per year from 1992 to 2022; Table S2: Analysis of 38 countries with more than five relevant publications in TMJ disc displacement.
