# Supplementary material for: A Bibliometric Analysis of Research on Temporomandibular Joint Disc Displacement from 1992 to 2022
Source: Healthcare (Basel). 2023 Jul 24;11(14):2108. doi: 10.3390/healthcare11142108 (PMC10379638; doi:10.3390/healthcare11142108)
Supplement: Supplementary file 1 [file healthcare-11-02108-s001.zip › Table S1.pdf]

Table S1. Number of publications and citations in TMJ disc displacement per year from 1992 to 2022.

Year	Publication	Citation
1992	22	7
1993	22	16
1994	21	40
1995	35	74
1996	34	144
1997	31	133
1998	40	226
1999	40	273
2000	47	339
2001	55	447
2002	54	541
2003	57	683
2004	52	590
2005	56	668
2006	53	858
2007	58	999
2008	47	1001
2009	57	1117
2010	56	1139
2011	50	1233
2012	68	1519
2013	74	1758
2014	64	1645
2015	64	1814
2016	75	1915
2017	74	1840
2018	97	2237
2019	104	2303
2020	129	3220
2021	124	3460
2022	122	3471
