# Supplementary material for: A Bibliometric Analysis of Research on Temporomandibular Joint Disc Displacement from 1992 to 2022
Source: Healthcare (Basel). 2023 Jul 24;11(14):2108. doi: 10.3390/healthcare11142108 (PMC10379638; doi:10.3390/healthcare11142108)
Supplement: Supplementary file 1 [file healthcare-11-02108-s001.zip › Table S2.pdf]

Table S2. Analysis of 38 countries with more than five relevant publications in TMJ disc displacement.

Rank	Country	Documents	Citations	Average Citations
1	USA	314	9853	31.38
2	Japan	293	5983	20.42
3	PRC	227	2520	11.10
4	Brazil	175	2617	14.95
5	Turkey	137	1727	12.61
6	Italy	103	1965	19.08
7	Germany	102	1917	18.79
8	South Korea	77	1074	13.95
9	Sweden	72	2544	35.33
10	Austria	72	1866	25.92
11	Canada	55	1773	32.24
12	Spain	52	965	18.56
13	Egypt	46	487	10.59
14	Netherlands	43	1297	30.16
15	Poland	33	278	8.42
16	India	29	132	4.55
17	Switzerland	27	537	19.89
18	Australia	27	465	17.22
19	Norway	25	785	31.40
20	Denmark	24	817	34.04
21	France	23	265	11.52
22	UK	22	496	22.55
23	Saudi Arabia	20	107	5.35
24	Croatia	19	143	7.53
25	Singapore	17	507	29.82
26	Romania	17	103	6.06
27	Finland	14	447	31.93
28	Chile	14	254	18.14
29	Israel	13	381	29.31
30	Portugal	11	270	24.55
31	Iran	11	94	8.55
32	Thailand	10	147	14.70
33	Belgium	9	269	29.88
34	Czech Republic	7	98	14.00
35	Greece	6	166	27.67
36	Slovakia	6	46	7.67
37	Yemen	5	169	33.80
38	Serbia	5	61	12.20
