# Supplementary figures and images for: A Bibliometric Analysis of Research on Temporomandibular Joint Disc Displacement from 1992 to 2022
Source: Healthcare (Basel). 2023 Jul 24;11(14):2108. doi: 10.3390/healthcare11142108 (PMC10379638; doi:10.3390/healthcare11142108)

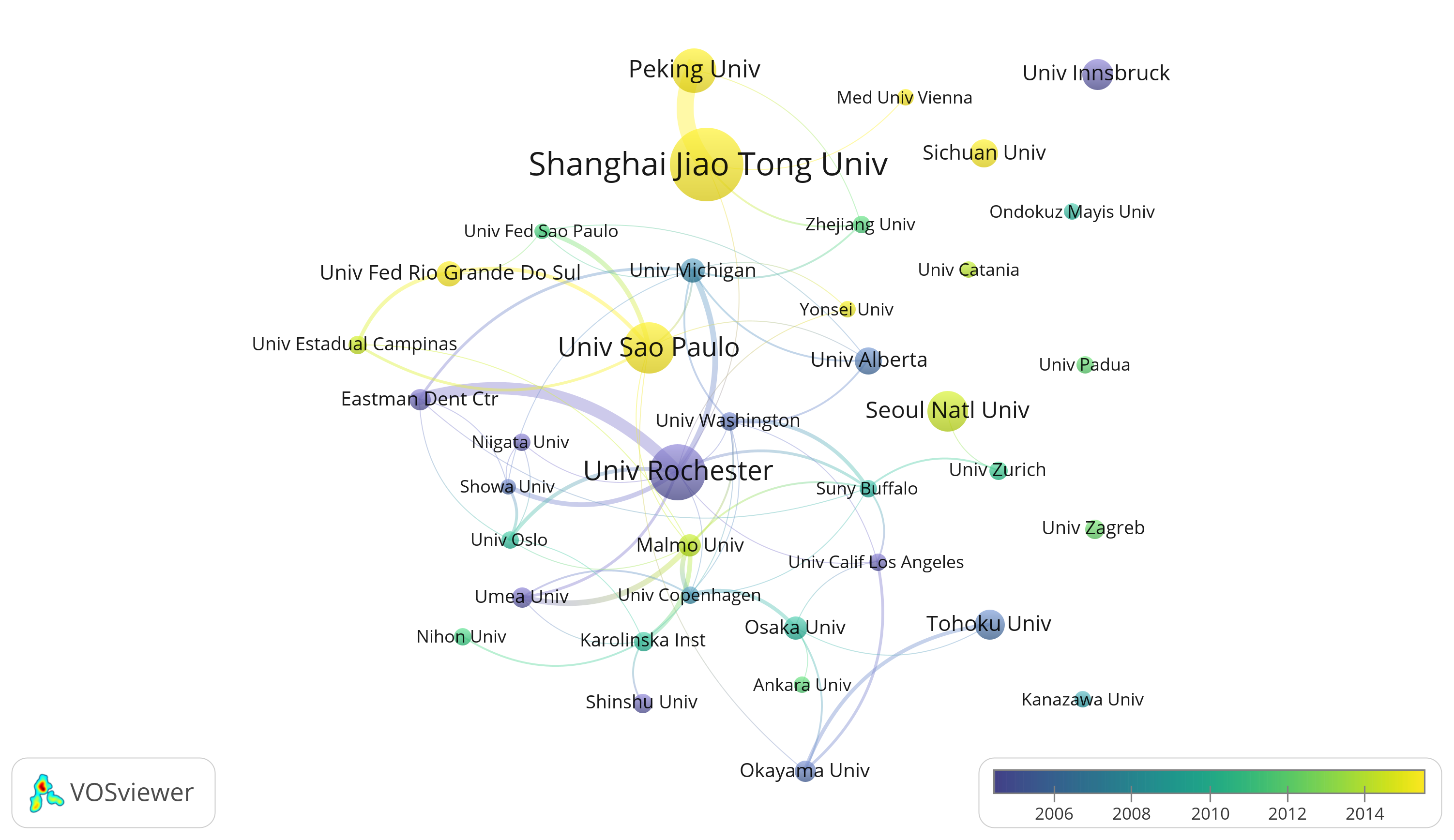

Supplement: Supplementary file 1 [file healthcare-11-02108-s001.zip › Figure S3.png]

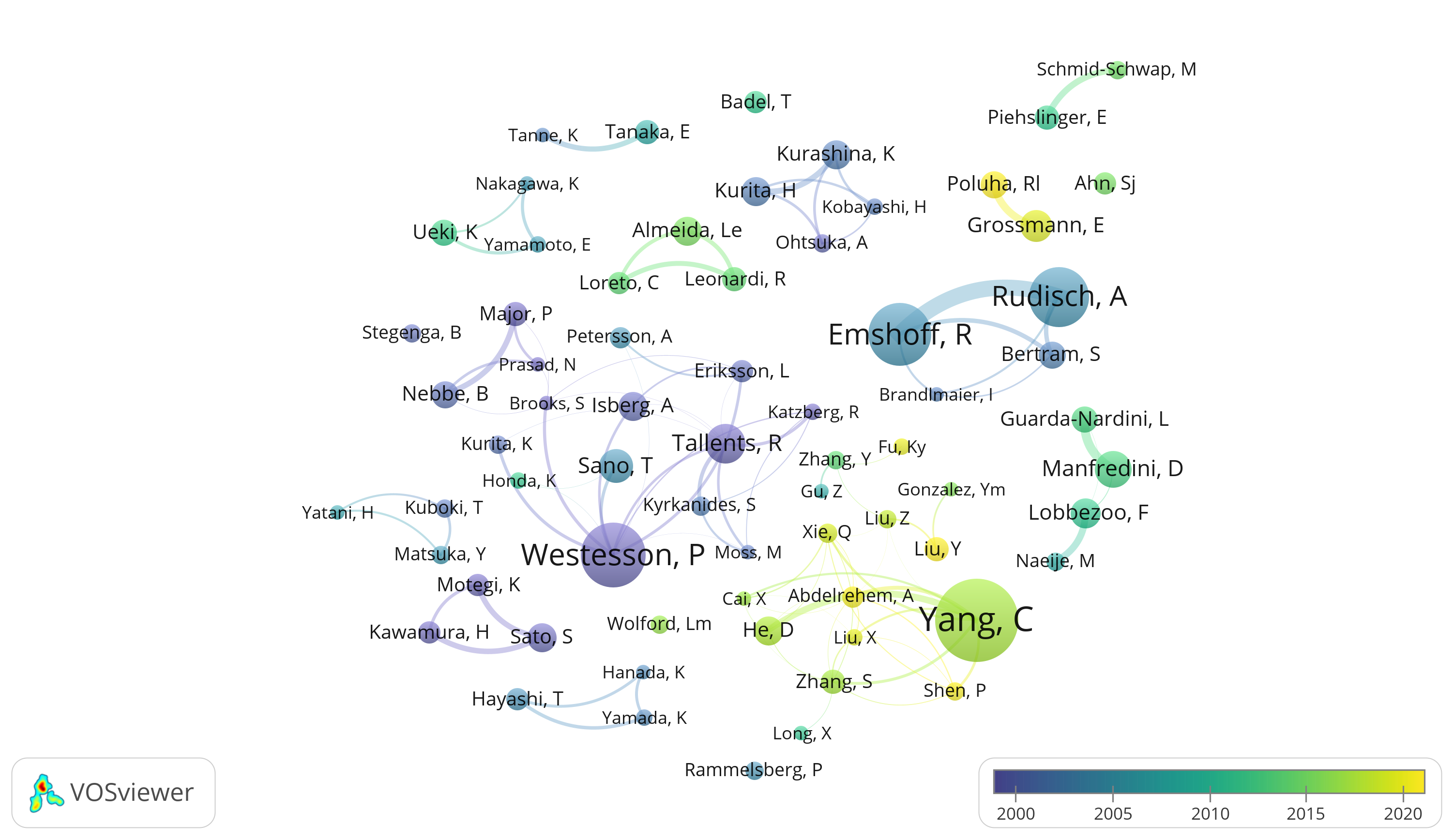

Supplement: Supplementary file 1 [file healthcare-11-02108-s001.zip › Figure S4.png]

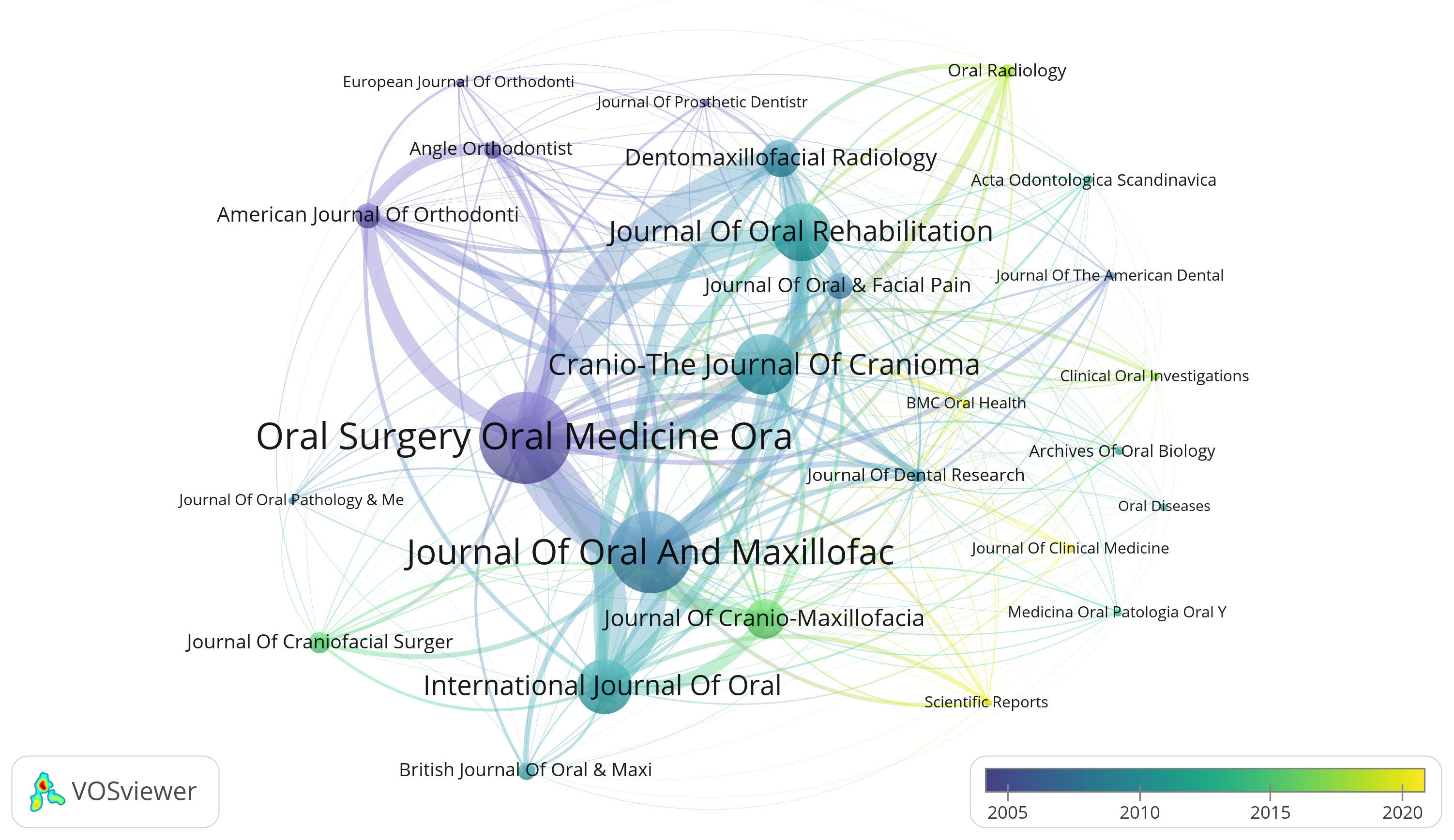

Supplement: Supplementary file 1 [file healthcare-11-02108-s001.zip › Figure S5.png]
